# Functional Ionic Liquid Modified Core-Shell Structured Fibrous Gel Polymer Electrolyte for Safe and Efficient Fast Charging Lithium-Ion Batteries

**DOI:** 10.3389/fchem.2019.00421

**Published:** 2019-06-12

**Authors:** Xiaoxia Liu, Yufei Ren, Lan Zhang, Suojiang Zhang

**Affiliations:** Beijing Key Laboratory of Ionic Liquids Clean Process, CAS Key Laboratory of Green Process and Engineering, Institute of Process Engineering, Chinese Academy of Sciences, Beijing, China

**Keywords:** lithium ion battery, fast charging, core-shell structure, nanofibrous separator, safety

## Abstract

Fast charging is of enormous concerns in the development of power batteries, while the low conductivity and lithium ion transference number in current electrolytes degraded the charge balance, limited the rate performance, and even cause safety issues for dendrite growth. Combine inorganic fillers and ionic liquid plasticizer, here in this paper we prepared a core-shell structured nanofibrous membrane, by incorporating with carbonate based electrolyte, a gel polymer electrolyte (GPE) with high conductivity, outstanding Li^+^ transference number was obtained. Notably, the Li/electrolyte/LiNi_0.6_Co_0.2_Mn_0.2_O_2_ (NCM622) half-cell with this composite electrolyte delivers a reversible capacity of 65 mAh/g at 20C, which is 13 times higher than that of with Celgard 2325 membrane. It also shows enhanced long-term cycle stability at both 3C and 5C for the suppression of lithium dendrite. This organic-inorganic co-modified GPE guarantees the fast charging ability and safety of LIBs, thus provides a promising method in high performance electrolyte design.

## Introduction

With the increasing demand for energy consumption and the continuous improvement for environmental protection, the development of sustainable and renewable energy is urgently needed currently. Lithium ion batteries (LIBs) have been widely used in the power battery, energy storage applications, portable electronic equipment and other fields, owing to its inherent advantages such as high energy density, high power density, long cycle life, no memory effect, and environmental friendliness (Gao et al., 2009; Goodenough and Kim, 2010; Etacheri et al., 2011; Scrosati et al., 2011; Lu et al., 2014a; Varzi et al., 2016; Tan et al., 2018). Nevertheless, high-capacity and high-power LIBs are facing more and more challenges such as high power output, rapid charge/discharge capability and safety under dynamic conditions (Zhu et al., 2013; Xie et al., 2014; Zhang J. et al., 2014; Li et al., 2018, 2019), and it is of particularly importance to investigate methods to guarantee the safety of LIBs with enhanced fast charge capability (Rui et al., 2016; Tan et al., 2018).

LIBs generally compose of anode, cathode, electrolyte, and packing material. The electrolyte, which including polymer separator and liquid electrolyte in commercial LIBs, mainly plays two key roles: blocking the direct contact between the electrodes to avoid internal electrical short circuit and acting as the Li^+^ transport passage (Tarascon and Armand, 2001; Xiao et al., 2009; Huang, 2010; Lee et al., 2014). Therefore, the electrolyte not only directly affects the LIB electrochemical performance, such as cycle stability and rate capability, but also, it has significant influence on the safety of LIBs. Most commercial LIBs adopt polyolefin [such as polyethylene (PE) and polypropylene (PP)] membranes (Huang, 2010; Kim et al., 2015; Shen et al., 2018) and LiPF_6_/carbonates electrolyte, based on which satisfying cycle performances have achieved, while the rate capability and safety, especially during quick charge, is yet to be enhanced.

The major issue in fast charging LIBs is the poor kinetics caused by the sluggish ion transport in the cell, which will probably lead to large polarization and low reversible capacity, furthermore, when the anode potential is lower than that of Li plating, dendrite will be generated and may short the cell thus cause safety concerns.

Electrolyte holds significant influence on both rate capability and safety, on one hand, ionic conductivity and Li^+^ transference number is the major factor to control the battery kinetics (Diederichsen et al., 2017), on the other hand, the separator, which blocks the direct contact between electrodes, should also work as a physical barrier to suppress the dendrite from shorting the battery (Jana et al., 2015; Shin et al., 2015; Zheng et al., 2018). Therefore, scientist made lots of efforts in electrolyte modification, including surface coating of the separator (Ghazi et al., 2017; Liu et al., 2017a,b; Zuo et al., 2017), doping inorganic nanoparticles in polymer to obtain composite electrolyte (Xiao et al., 2012; Chen et al., 2017; Kim et al., 2017; Wang et al., 2017; Shen et al., 2018), and electrostatic spinning to get nanofibrous membrane (Choi et al., 2003; Wu et al., 2015; Ma et al., 2017; Cheng et al., 2018). Thereinto, the nanofibrous membrane has large specific surface area, three-dimensional (3D) porous structure and high electrolyte uptake ability, on which the rate capability can be enhanced (Xiao et al., 2015; Liang et al., 2016; Park et al., 2017). What's more, functional groups can be easily introduced in the electrospining process, thereby endows the electrolyte with other properties, such as dendrite suppression (Lu et al., 2015a,b; Cheng et al., 2018; Deng et al., 2018), flame retardant (Lu et al., 2017; Jia et al., 2018; Sun et al., 2018), et al. Poly(vinylidene fluoride-co-hexafluoropropene) (PVDF-HFP) is considered as a potential polymer matrix for electrolyte not only as it has a low crystallinity which could promotes rapid ion conduction (Ali et al., 2018; Zhao et al., 2019), but also, it has high dielectric constant and low surface energy that may promote the compact deposition of metal Li (Lopez et al., 2018). It is reported the ionic conductivity of PVDF-HFP-based polymer electrolytes is up to 3.9 mS/cm (Xiao et al., 2012).

Ionic liquid modified gel polymer electrolytes (IL-GPE) have attracted much attention due to their good thermal stability and mechanical properties. Singh et al. studied imidazolyl ionic liquids and found that the EMIMFSI-based GPEs have excellent electrochemical stability, good compatibility and thermal stability(Singh et al., 2018). Guo et al. prepared the PVDF-HFP-LiTFSI/SiO_2_/EMITFSI GPE with high thermal stability and good electrode compatibility (Guo et al., 2018). However, IL-GPE also has some shortcomings, such as lower ionic conductivity and lithium ion mobility (Zhou et al., 2015). In this work, a piperidine ionic liquid, 1-methyl-1-propylpiperidinium chloride (PPCl), and Li_2_SiO_3_ (LSO) nanoparticles were introduced into the PVDF-HFP matrix *via* coaxial electrospinning technology. The as prepared membrane has high porosity and electrolyte uptake, remarkable ionic conductivity, and outstanding electrochemical performance especially in quick charging. Commercial NCM622 cathode adopting this IL-GPE delivers a high reversible capacity of 65 mAh/g in 20C rate charge/discharging, which is 13 times higher than that of the cell adopting Celgard 2325 membrane.

## Experimental Section

### Materials

Poly (vinylidene fluoride-co-hexafluoropropene) (PVDF-HFP, Mw. ~455,000), 1-methylpiperidine (97%), and (3-chloropropyl) trimethoxysilane (98%) were provided by Sigma-Aldrich. Li_2_SiO_3_ (LSO, 99%, Strem Chemicals), N, N-dimethylformamide (DMF, 99.5%, Beijing Chemical Works), LiNi_0.6_Co_0.2_Mn_0.2_O_2_ (NCM622, Beijing Dangsheng Material Technology Co., Ltd.), Super P (Imerys Graphite & Carbon), Polyvinylidene difluoride (PVDF, Solvay 5130), N-Methyl-2-pyrrolidone (NMP, 99.0%, Sinopharm Chemical Reagent Co., Ltd.), lithium tablet (Li, Tianjin Zhongneng Co., Ltd.), and polypropylene (PP, Japan Ube) were commercially available and used without further purification.

### Preparation of the GPEs

**Scheme 1:**
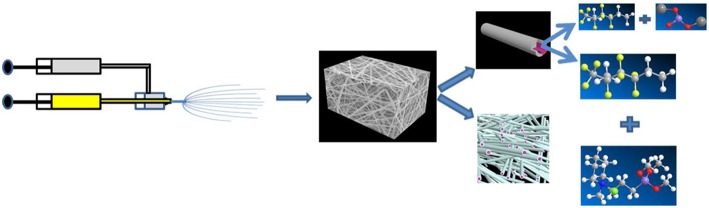
Illustration of the preparation of the PHP@PHL membrane (Zhou et al., 2013).

The ionic liquid PPCl was synthesized according to the procedure reported before (Lu et al., 2012; Korf et al., 2014; Cheng et al., 2018), its structure and purity was also testified in our previous work (Xu et al., 2018). The core-shell structured 3D porous nanofiber membrane was prepared by the coaxial electrospinning technique on an ET-2535H machine (Ucalery Tech Inc., China), as shown in Scheme 1. To make composite nonwoven membrane, 80% (wt., similarly hereinafter) DMF was adopted in all the spinning solutions. The slurry for core spinning was prepared by mixing PPCl and PVDF-HFP in DMF solvent, wherein the weight ratio of PPCl: PVDF-HFP: DMF was fixed at 1:19:80. Correspondingly, the slurry for shell spinning was prepared by mixing LSO and PVDF-HFP in DMF solvent with a ratio of 2:18:80. The coaxial electrospinning equipment mainly contained a variable positive voltage of 15 kV and a negative voltage of −2kV, two syringe pumps, a spinneret consisting of two chambers and a collector. The pumped speed of the shell and core solutions supply were fixed at 0.25 and 0.15 mL/h, respectively, the distance between the needle tip and aluminum foil collector was 13 cm. The as prepared membrane has a thickness of 50 ± 5 μm, and was entitled by PHP@PHL. Accordingly, PVDF-HFP, PVDF-HFP-PPCl (PHP), and PVDF-HFP-LSO(PHL) nanofiber membrane was prepared by mixing PVDF-HFP in DMF (20:80), PPCl, and PVDF-HFP in DMF (1:19:80) or LSO and PVDF-HFP in DMF (2:18:80), respectively. The as-prepared nonwoven fiber membranes were cut into disc with a diameter of 16 mm, which were then dried in a vacuum oven at 60°C for 20 h to remove the residual solvent. In the end, the membranes were filled and swollen in a liquid electrolyte, 1.2 M LiPF_6_ in ethylene carbonate (EC) and ethyl methyl carbonate (EMC) (3:7, weight ratio), for 30 min in an argon filled glove box to obtain the relevant GPEs.

### Characterization of the Membranes and GPEs

The thickness of various films was recorded by measuring membrane apparatus (CH1ST, Shanghai Milite Precise Instrument Co., Ltd., China), and morphology of the membrane was studied by field-emission scanning electron microscopy (FE-SEM, JSM-7001F, JEOL, Japan). The field emission transmission electron microscopy (TEM, JEOL, JEM-2100) was used to test the core-shell structure of PHP@PHL nanoporous fiber membrane. The surface chemical composition of PHP@PHL was analyzed by X-ray photoelectron spectroscopy (XPS, ESCALAB 250Xi, Thermo Fisher Scientifec, America). Differential scanning calorimetry (DSC, Mettler-Toledo, Switzerland) was carried out to analyze the thermal behavior of all kinds of membranes. Samples were put into aluminum pans and the test temperature was set from 50 to 250°C with a heating rate of 5°C/min, under N_2_ atmosphere. The porosity of various films was measured by soaking n-butanol for 2 h, then calculated using Equation (1): P = (m_b_/ρ_b_)/(m_b_/ρ_b_ + m_a_/ρ_a_) ×100%, where m_a_ and m_b_ are the weights of separators and n-butanol, ρ_a_ and ρ_b_ are the density of separators and n-butanol, respectively (Xiao et al., 2012; Zhou et al., 2013). In an argon filled glove box, the electrolyte uptakes were analyzed by the mass difference of separators before and after soaking in electrolyte for 30 min and then calculated using Equation (2): EU = (W–W_0_)/W_0_ ×100%, in which W_0_ and W are the weights of the films before and after immersing in the liquid electrolyte, respectively. Ten samples are tested to measure the electrolyte uptake and porosity of each membrane. Wettability of the separators were researched by contact angle measurements (DSA 100S, Germany KRUSS).

The various GPEs' effective ionic conductivities (σ) were calculated using Equation (3): σ = d/(R_b_ × S), where d is the thickness of the film, S is the area and R_b_ is the bulk impedance acquired by electrochemical impedance spectroscopy (EIS) in Stainless Steel (SS)/electrolyte/SS symmetric cells. The bulk resistances (R_b_) were investigated by a CHI660E electrochemical workstation with a frequency range of 0.01–10^5^Hz and an amplitude of 5 mV at room temperature. The lithium ion transference numbers (t_*Li*_^+^) of different electrolytes were tested using chronoamperometry (CA) and EIS (both by CHI660E), and then calculated using Equation (4): t_*Li*_^+^ = I_S_(Δv–I_0_R_0_)/I_0_(Δv–I_S_R_S_) (Li et al., 2018), where I_0_ and I_S_ are the initial and steady state currents obtained by CA testing of lithium symmetrical cell, R_0_ and R_S_ are the interfacial impedance before and after polarization, and ΔV (10 mV) is the applied voltage difference (Yang et al., 2014; Zhang F. et al., 2014). The electrochemical performances were researched in coin cells with lithium foil anode and LiNi_0.6_Co_0.2_Mn_0.2_O_2_ (NCM622) cathode. The interfacial stability between the electrode and electrolytes was investigated using EIS after standing for 1, 10, 20, and 30 days, respectively. The long term cycling stability, as well as rate performances were tested in a voltage range between 2.8 and 4.4 V with different C rates by LAND battery cycle system (Wuhan Blue Electric Co., LTD, China).

## Results and Discussion

### Morphologies of the Membranes

**Figure 1:**
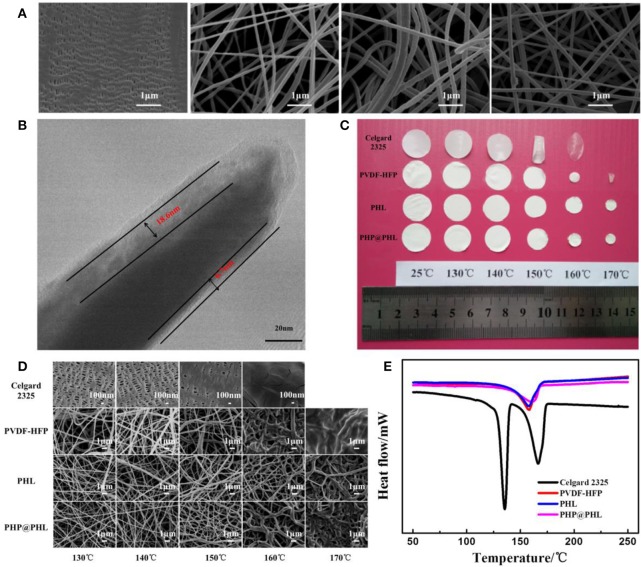
**(A)** SEM images of the various separators, Celgard 2325, nanofibrous PVDF-HFP, PHL, and PHP@PHL; **(B)** TEM micrograph of the PHP@PHL fibers; **(C)** Picture of Celgard 2325, PVDF-HFP, PHL, and PHP@PHL separators and their macroscopic changes after heat treatment at different temperatures, **(D)** SEM pictures of Celgard 2325, PVDF-HFP, PHL, and PHP@PHL separators and their morphologies after heat treatment under different temperatures, **(E)** DSC curves of Celgard 2325, PVDF-HFP, PHL, and PHP@PHL separators.

The morphologies of the Celgard 2325 (which is a PP/PE/PP trilayer separator), nanofibrous PVDF-HFP, PHL, and PHP@PHL membranes, as well as microscopic changes after the thermal stability analyze in 130–170°C temperature range, were tested by using SEM, as shown in Figures 1A,D. The Celgard 2325 membrane has tensile holes in the lamellar matrix, but the electrostatic spinning films show 3D porous structure with 80–160 nm diameter nanofiber which interlacing with each other to form interconnected networks. The energy dispersive X-ray spectroscopy (EDS) data shown in Figure S1 gives the elemental distribution in PHL and PHP@PHL complex films. As shown in Figure S1a, Si and O are uniformly distributed in the membrane, which proves that Li_2_SiO_3_ is evenly dispersed in the polymer matrix. EDS results of PHP@PHL in Figure S1b shows little signal of N or Cl as the content of PPCl is pretty low (<2%). TEM image of PHP@PHL nanofiber further proved its core-shell structure, and the inorganic nanoparticle Li_2_SiO_3_ is dispersed in the out layer of the PVDF-HFP matrix, with the thickness of several to tens of nanometers as shown in Figure 1B. The XPS spectra in Figure S2 detected no Cl element on the surface of PHP@PHL nanofiber, which further proves that the PPCl ionic liquid was fully encapsulated by the out PHL layer.

The thermal stability of various membranes was investigated by storing them in an air circulation oven at a series of temperatures between 130 and 170°C, each for 20 min. Figure 1C shows that Celgard 2325 begins to curl for the melting of PE and molecular tanglement of polyolefin at about 135°C, it turns transparent at 160°C for the melt of PP, meanwhile PVDF-HFP shrink at 150°C and melt at 170°C. However, the PHL and PHP@PHL keeps their porous structure even at 170°C. It can be concluded that the inorganic nano-particle, Li_2_SiO_3_, in the shell structure significantly improved the thermal stability of the separator, which may potentially contribute in enhancing the safety of the battery. In order to further clarify the morphology changes of the films after heat treatment, FE-SEM pictures of the four separators are given in Figure 1D for microscopic description. Celgard 2325 was fully shutdown at 160°C, and PVDF-HFP nanofibrous films melt under 170°C, but the PHL and PHP@PHL films still keeps certain amount of pores remain in 170°C (Figure 1D). From the microscopic point of view, the core-shell structure nanofibrous membrane expresses excellent thermal stability, hence makes it have distinguished heat resistance and advance the safety of lithium battery under high temperature condition. According to DSC test results of four separators, membranes based on PVDF-HFP shows better thermal stability than that of Celgard 2325, and the core-shell structured PHP@PHL, released the least heat even on melting (Figure 1E) (Kang et al., 2016; Wang et al., 2019).

**Figure 2:**
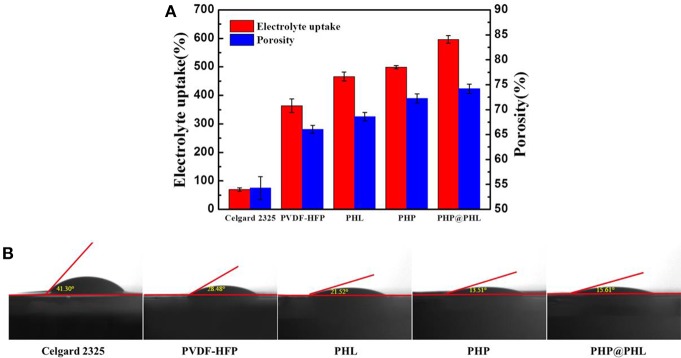
Electrolyte uptake and porosity **(A)**, as well as the contact angle images with liquid electrolyte droplet **(B)** of the Celgard 2325, PVDF-HFP, PHL, PHP, and PHP@PHL separators.

Porosity of the membranes are tested and calculated by Equation (1) as shown in Figure 2A, it's about 54.4, 66.1, 68.5, 72.5, and 74.0% for Celgard 2325, PVDF-HFP, PHL, PHP, and PHP@PHL, respectively. Obviously, fibrous separators have higher porosity than Celgard 2325, which may contribute in electrolyte uptake and thus enhance the rate capability. Contact angle of liquid electrolyte to various membranes were also studied and shown in Figure 2B. The contact angle of Celgard 2325, PVDF-HFP, PHL, PHP and PHP@PHL is 41.30, 28.48, 21.52 13.51, and 15.61°, respectively. All fibrous membranes showed smaller contact angle with electrolyte for the high dielectric constant of PVDF-HFP (Lopez et al., 2018), besides, they also beneficial from the fibrous structure. Smaller contact angle indicated that the PHP@PHL composite nanofibrous membrane has better affinity and is easier to be wetted by the liquid electrolyte, thus better rate capability might be obtained.

**Figure 3:**
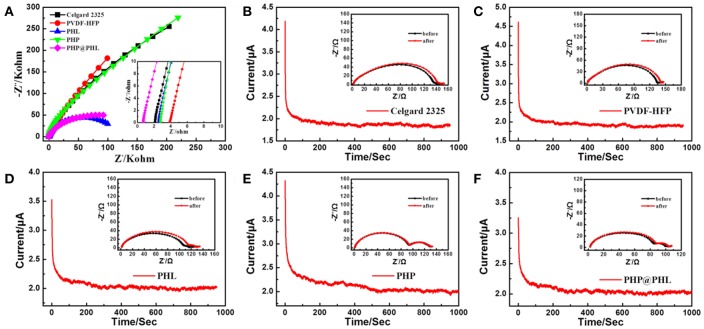
**(A)** Nyquist plot of AC impedance measurements (SS/separator/SS) of the Celgard 2325, PVDF-HFP, PHL, and PHP@PHL separators; Lithium ion transference numbers of **(B)** the Celgard 2325, **(C)** PVDF-HFP, **(D)** PHL, **(E)** PHP, and **(F)** PHP@PHL membranes as demonstrated by CA polarization curves and EIS plots before and after polarization.

The effect ionic conductivity of the five separators was probed by EIS test at ambient temperature and calculated by Equation (3) as shown in Figure 3A. R_b_ obtained from EIS data is 2.1105, 3.8473, 2.5165, 3.3936, and 0.6801 Ω for Celgard 2325, PVDF-HFP, PHL, PHP, and PHP@PHL, respectively, and the corresponding effect ionic conductivity are 0.63, 0.64, 1.03, 1.02, and 4.05 mS/cm, respectively. Consistent with the results of porosity and wettability, this data further guaranteed the enhanced rate capability in cells.

The lithium ion transference number (t_*Li*_^+^) of Celgard 2325 and the as-prepared membranes with liquid electrolyte was tested and calculated via Equation (4) as shown in Figures 3B–F, where the corresponding t_*Li*_^+^ are 0.42, 0.39, 0.56, 0.45, and 0.62, respectively. t_*Li*_^+^ reduced from 0.42 to 0.39 when Celgard 2325 was replaced by PVDF-HFP, which proves that the polymer itself in fact has little contribute in Li^+^ transference. The addition of Li_2_SiO_3_ enhanced the number to 0.56, which demonstrated that the inorganic nanoparticle may facilitate the desolvation of Li^+^. While t_*Li*_^+^ of PHP is only 0.45, slightly higher than that of Clegard 2325 and PVDF-HFP, the reason lays on that although PPCl was mixed with PVDF-HPF and formed a membrane, but the Cl anion could still partly dissociated into the electrolyte thus influence the transportation of Li^+^ (Xu et al., 2018). PHP@PHL shows the highest t_*Li*_^+^ among the five electrolytes for the synergistic effect of LSO and PPCl, encapsulated by the PHL layer, the Cl anion could not influence the Li^+^ transport but adjust the polarization of the separator polymer matrix. Therefore, PHP@PHL nanofiber membrane prominently improves the t_*Li*_^+^, which may contribute in reduces anion aggregation near the electrode surface, thus reduces the concentration polarization and stabilizes the electrochemical deposition of lithium ions, improving the safety of LIB.

The film thickness, electrolyte uptake, porosity, ionic conductivity, and lithium ion transference number of the Celgard 2325, PVDF-HFP, PHL, and PHP@PHL separators summarized in Table 1. It is worth noting that the electrolyte uptake of PHP@PHL was about 597%, which is much higher than that of Celgard 2325 (70%), PVDF-HFP (366%), PHL (466%), and PHP (498%) and further contribute in the better rate capability when adopting in the LIBs. PHP@PHL composite membrane demonstrated the highest porosity and electrolyte uptake ability, the smallest electrolyte contact angle, and there are several factors that contribute to its remarkable performances. Firstly, the molecular structure of PVDF-HFP, as well as the 3D nanofibrous morphology facilitated the electrolyte uptake, for its high polarity and the nanoporous structure. Secondly, Li_2_SiO_3_ and PPCl reduced the order of molecular arrangement, which produced more chances for the electrolyte to be taken. Moreover, PPCl also worked as a plasticizer which has perfect affinity with the electrolyte, on which the contact angle is also reduced. Therefore, PHP@PHL shows the best physical performances. We also compare the ionic conductivity and the t_*Li*_^+^ with some of the previous publications as listed in Table 2, obviously, by the synergistic effect of LSO and PPCl, lithium ion transport efficiency was strongly enhanced.

**Table 1:** The film thickness, electrolyte uptake, porosity, effective ionic conductivity, and lithium ion transference number of Celgard 2325, PVDF-HFP, PHL, PHP, and PHP@PHL separators.

	**Films thickness (μm)**	**Electrolyte uptake (%)**	**Porosity (%)**	**Conductivity (mS cm^**−1**^)**	***t_***Li***_*^**+**^**
Celgard 2325	25 ± 1	70 ± 3	54.4 ± 1.1	0.63	0.42
PVDF-HFP	50 ± 5	366 ± 12	66.1 ± 0.4	0.64	0.39
PHL	50 ± 5	466 ± 8	68.5 ± 0.4	1.03	0.56
PHP	50 ± 5	498 ± 3	72.5 ± 0.5	1.02	0.45
PHP@PHL	50 ± 5	597 ± 8	74.0 ± 0.5	4.05	0.62

**Table 2:** Ionic conductivity and t_*Li*+_ in previous relevant works.

**Type of GPE**	**Conductivity σ (mS/cm)**	***t_***Li***_*^**+**^**	**References**
PVDF-HFP@Al_2_O_3_ GPE	1.24	–	Shen et al., 2018
40 wt% IL GPE	0.15	0.39	Singh et al., 2018
PSA@PVDF-HFP	1.97	–	Zhou et al., 2013
PMMA/PVDF-HFP	1.31	–	Zhang J. et al., 2014
MS5 based GPE	3.2	0.62	Li et al., 2018
Gel PVDF–NWF	0.3	–	Zhu et al., 2013
SiO_2_@Li^+^ doped PVDF–HFP	3.9	0.44	Xiao et al., 2012
NanoIL GPE	0.64	0.6	Cheng et al., 2018
PHP@PHL	4.05	0.62	This work

**Figure 4:**
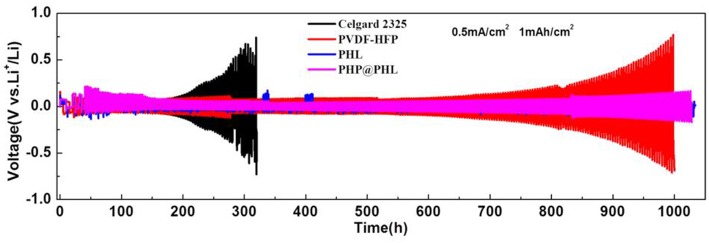
Galvanostatic cycling performances of symmetric lithium cells with the Celgard 2325, PVDF-HFP, PHL, and PHP@PHL separators at a current density of 0.5 mA/cm^2^ at 25°C.

Galvanostatic cycling measurements in Li/electrolyte/Li symmetrical cells can probed into lithium plating/stripping process and analyze the interfacial stability between the electrolyte and lithium electrode. The charge/discharge cycling test was performed at a fixed current density of 0.5 mA/cm^2^ with a total capacity of 1 mAh/cm^2^ and the results are shown in Figure 4. As can be seen from the Celgard 2325 voltage-time profile, it exhibits a gradual increase in hysteresis (overpotential between Li deposition and dissolution) as the time increases, and the voltage increases to 0.65 V after 300 h. In other words, the SEI film, or dead lithium is thickening with cycling, which means an out-off-balance lithium plating/stripping. The voltage-time profiles of PVDF-HFP, PHL, and PHP@PHL electrolytes were much stable compared with that of Celgard 2325. In particular, although the t_*Li*_^+^ of PVDF-HFP is lower than that of Celgard 2325, while it turns quite stable in the initial 500 h owing to the fibrous structure of the membrane which could balance the Li^+^ flux adjacent to the electrode. PHL presents the least hysteresis after 1,000 h plating/stripping, while as it shows several abnormal convex during the cycling, therefore, we consider that the PHP@PHL cell demonstrated the best stability. As discussed before, the enhanced performance of PHP@PHL can be attributed to several reasons, firstly, the nanofibrous structure of PVDF-HFP balanced the Li^+^ flux and suppressed the mossy deposition of Li; secondly, the synergistic effect of LSO and PPCl reduce the crystallinity of the polymer matrix, thus enhanced the Li^+^ transportation efficiency; lastly, Cl anion from the IL may partly be involved in the formation of SEI, which further enhance the cycle stability of the symmetric cell. Hence, the formation of Li dendrites is inhibited and the speed of dendrite growth is reduced as well. These results manifest that using 3D porous electrostatic spinning nanofiber membrane could effectively lower the hysteresis, stabilize cycling behavior and elongate the cell lifetime.

The batteries tested with the above four electrolytes were then disassembled in an argon-protected glove box, the electrodes were cleaned with diethyl carbonate (DEC) and dried strictly, and then put into a vacuum transfer box before FE-SEM analysis. The experimental results are shown in Figure S3. For the symmetrical lithium battery assembled with Celgard 2325 after galvanostatic charge/discharge cycle for 300 h, the FE-SEM diagram indicates that a lot of clavate-shaped lithium dendrite is generated on the surface of the lithium electrode. These dendrites will eventually penetrate the separator, causing short circuit and safety problems. By contrast, the symmetrical lithium battery assembled with the electrostatic spinning films, PVDF-HFP, PHL, and PHP@PHL, even cycled for 1,000 h under the same current density, shows much smoother surface with smooth edges, which was not easy to penetrate the separators, greatly improved the safety of LIBs. The electrospinning separators have large specific surface area, which could balance the local current density on the electrode surface, thus inhibited the formation and growth of lithium dendrite. Therefore, the core-shell nanofiber separator prepared by coaxial electrostatic spinning can significantly improve the safety performance of LIB.

**Figure 5:**
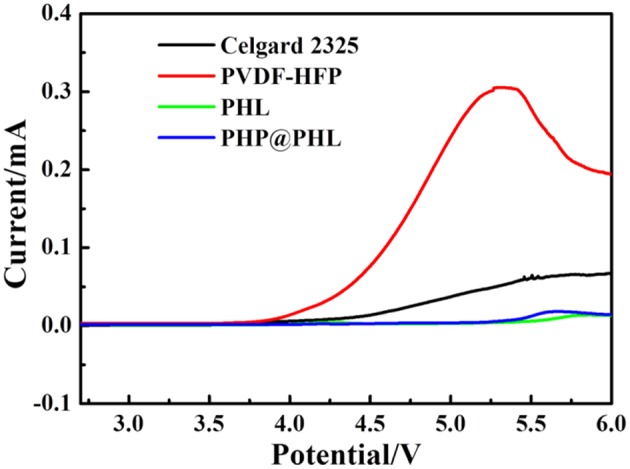
Linear sweep voltammograms of the four electrolytes at a scan rate of 0.5 mV/s (Li/electrolyte/SS).

Linear sweep voltammetry (LSV) was used to investigate the electrochemical windows of the electrolytes with a scan rate of 0.5 mV/s over the range of 2.8–6 V in Li/electrolyte/SS battery, and the results are shown in Figure 5. The oxidation potentials of the Celgard 2325, PVDF-HFP, PHL, and PHP@PHL electrolyte is 4.27, 3.93, 5.5, and 5.45 V (vs. Li^+^/Li), respectively. As all these membranes are plasticized by the same electrolyte, LiPF_6_/EC+EMC, therefore, most parasitic reactions should origin from the membrane. Therefore, it can be concluded that both LSO and PPCl could prevent PVDF-HFP from being oxidized and effectively enhance the electrochemical stability of composite membrane, which is pivotal for practical application. No obvious decomposition of Li/PHP@PHL/SS battery was observed below 5.45 V, which manifested PHP@PHL may potentially be able to be coupled with high voltage cathode materials, such as NCM622.

**Figure 6:**
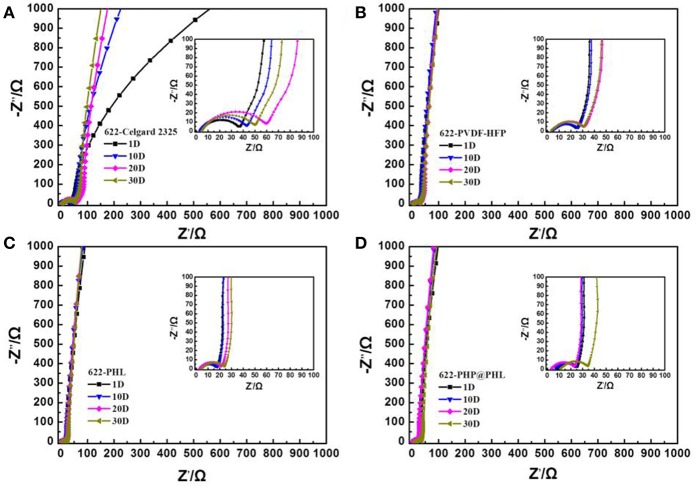
Impedance evolution of the NCM622/Li half-cells with **(A)** the Celgard 2325, **(B)** PVDF-HFP, **(C)** PHL, and **(D)** PHP@PHL membranes at open circuit potential as a function of storage time at 25°C for 1, 10, 20, and 30 days.

The interfacial stability between the electrode and the electrolyte is fatal for long-term cycle stability and rate performance, therefore, the separators are adopted and Li/electrolyte/NCM622 half-cells were assembled to further test their compatibility with the electrode materials. Static EIS tests were performed in 1, 10, 20, and 30 days after cell assembled at room temperature and the data was deal with an equivalent circuit fitting (Figure S4), the results are shown in Figure 6 and Table S1. R_b_ is corresponding to the bulk resistance originated from electrolyte and other cell components, R_1_ and R_2_ can be assigned to the interface resistance between electrolyte and anode or cathode, respectively. R_2_ is smaller than R_1_ because the battery is never charged after assembly and is left standing, therefore, little reaction would happen in the storage process. The cell with PHP@PHL shows the most stable R_1_ value among the four cells, it is 18.32 Ω after 30 days of storage, while it is 61.40, 35.45, and 35.42 Ω for Celgard 2325, PVDF-HFP and PHL, respectively, which indicates that PHP@PHL has the best interfacial compatibility with Li among these electrolytes (Cheng et al., 2018). Its smallest increase in interface resistance is mainly attributed to the following reason: firstly, PVDF-HFP has higher dielectric constant than PP or PE, which endows it with better Li compatibility (Lopez et al., 2018); secondly, the introduction of Li_2_SiO_3_ partially suppresses the decomposition of LiPF_6_ and carbonate solvents during long term storage (Fu et al., 2017); and the third, the slow dissociation of PPCl would generate some Cl anion, which would react with Li to form a more stable SEI component, LiCl, thus further stabilized the electrode/electrolyte interface and reduce its resistance (Lu et al., 2014b).

**Figure 7:**
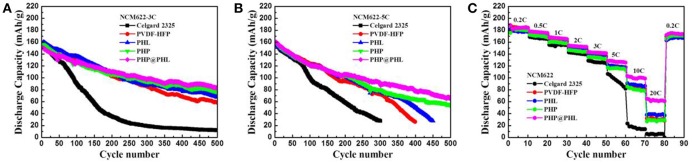
Long-term cycling stability of the NCM622/Li cells with different membranes (Celgard 2325, PVDF-HFP, PHL, PHP, and PHP@PHL) at 3C rate **(A)**, 5C rate **(B)**, and their rate capability **(C)**.

Electrochemical performances of the above mentioned GPEs were tested in Li/electrolyte/NCM622 half cells by different C rates. Figure S5 shows the 0.5C rate performance of Celgard 2325 and PHP@PHL, we can see that the cells delivers quite similar capacity in the initial 300 cycles, both cells kept more than 100 mAh/g reversible capacity in 500 cycles, which proves that the cathode material works well in this electrolyte system. It also can be notice that the Celgard one shows lower coulombic efficiency after about 350 cycles, which might cause by the mossy Li deposition after long term striping/plating. High rate tests were performed to testify the performance of nanofibrous GPEs. Figures 7A,B show the long term cycle performances of the Li/electrolyte/NCM622 half cells at 3C and 5C, respectively. Obviously, the reversible capacity and cycle stability of PHP@PHL system were significantly improved at these C rates. The initial discharge capacities of Celgard 2325, PVDF-HFP, PHL, PHP, and PHP@PHL were 156.6, 160.6, 161.4, 156.5, and 150.6 mAh/g at 3C rate, respectively. It drops sharply to 15.8 mAh/g after 300 cycles for the Celgard 2325 one, while that of PVDF-HFP, PHL, PHP, and PHP@PHL maintained at 85.3, 87.9, 84.1, and 98.5 mAh/g, respectively. The initial discharge capacities of Celgard 2325, PVDF-HFP, PHL, PHP, and PHP@PHL at 5C rate were 156.0, 156.0, 156.2, 158.7, and 160.2 mAh/g, respectively. It declined dramatically to 27.8 mAh/g after 300 cycles for Celgard 2325, and that of PVDF-HFP and PHL were also significantly reduced to 26.3 and 27.2 mAh/g after 400 cycles, respectively. But the one with PHP@PHL remained 78.5 mAh/g even after 500 cycles. The rate performances (from 0.2 to 20C) of Li/electrolyte/NCM622 half-cells are shown in Figure 7C. It can be seen that the difference between the electrospining GPEs and the Celgard separator was not obvious under low current density, but the diversity significantly improved under large current density, especially for PHP@PHL. The Li/electrolyte/NCM622 half-cell with PHP@PHL can still deliver a reversible capacity of 65 mAh/g at 20C, while that of Celgard 2325, PVDF-HFP, PHL, and PHP were 5, 30, 38, and 29 mAh/g, respectively, which means the PHP@PHL delivers 13 times higher capacity than that of Celgard 2325. Charge-discharge profiles of the Li/electrolyte/NCM622 half-cells with Celgard 2325 and PHP@PHL at different C rates were compared in Figure S6. It can be easily conclude from these results that the as prepared core-shell structure PHP@PHL nanofibrous electrolyte has the best electrochemical performances, which can realize safe and efficient quick charging, thus has great potential in the application of high-power density LIBs.

## Conclusions

In summary, a core-shell structured nanofibrous membrane with PPCl ionic liquid plasticizer in the core and inorganic nano-particle Li_2_SiO_3_ as the filler in shell, PHP@PHL, was prepared by a facile coaxial electrospining method. The electrolyte uptake, porosity, ionic conductivity and lithium ion transference number of PHP@PHL nanofiber membrane were about 597%, 74.0%, 4.05 mS/cm, and 0.62, respectively. By the introduction of PPCl and Li_2_SiO_3_, ionic conductivity of the electrolyte increases by an order of magnitude and wettability between the separator and liquid electrolyte also significantly improved compared to commercial Celgard 2325 or even electrospining PVDF-HFP separator. In addition, it has good thermal stability, low interfacial impedance and wide electrochemical window. Symmetrical lithium cells with PHP@PHL demonstrated excellent plating/stripping cycling stability for about 1,000 h without short-circuit, which is 5 times longer than that of Celgard 2325. Moreover, the PHP@PHL electrolyte presents outstanding rate capability, it delivers a reversible capacity of 65 mAh/g at 20C compared to the 5 mAh/g in the case of Celgard 2325. The long term cycle performance was also significantly improved, as demonstrated in 3C and 5C. The core-shell structured nanofibrous membrane, in which different fillers or plasticizers could be used thus different functions can be realized in one single membrane, provides and effective method to enhance the overall performances of LIBs.

## Data Availability

The raw data supporting the conclusions of this manuscript will be made available by the authors, without undue reservation, to any qualified researcher.

## Author Contributions

SZ helps to improve ideas and experimental platform. LZ helps to solve some problems of experience. XL helps to do mainly works. YR helps to do any experience.

### Conflict of Interest Statement

The authors declare that the research was conducted in the absence of any commercial or financial relationships that could be construed as a potential conflict of interest.
