# Supplementary material for: Functional Ionic Liquid Modified Core-Shell Structured Fibrous Gel Polymer Electrolyte for Safe and Efficient Fast Charging Lithium-Ion Batteries
Source: Front Chem. 2019 Jun 12;7:421. doi: 10.3389/fchem.2019.00421 (PMC6581669; doi:10.3389/fchem.2019.00421)
Supplement: Supplementary file 1 [file Data_Sheet_1.pdf]

Supporting Information

Functional ionic liquid modified core-shell structured fibrous gel polymer electrolyte for safe and efficient fast charging lithium-ion batteries

Experimental Section

Synthesis and characterization of PPCl:

The PPCl was synthesized by a facile one step way. 1-methylpiperidine (0.05mol) and (3-chloropropyl) trimethoxysilane (0.06mol) with the mole ratio of 1:1.2 was reacted in DMF (20ml) in a round bottom flask (100ml) under N_2 protection. The temperature was set to 80℃ with continuous agitation, and it takes 2-3 days to finish the reaction. After washed by the anhydrous ether for 3-5 times, treated with the rotary evaporator, and finally dried at 60℃ for 1 day in the vacuum oven, the final product, a yellow powder was obtained.(Cheng et al., 2018; Korf, Lu, Kambe, & Archer, 2014; Lu, Moganty, Schaefer, & Archer, 2012) The product was stored in the glove box for the subsequent tests and uses.

(a)

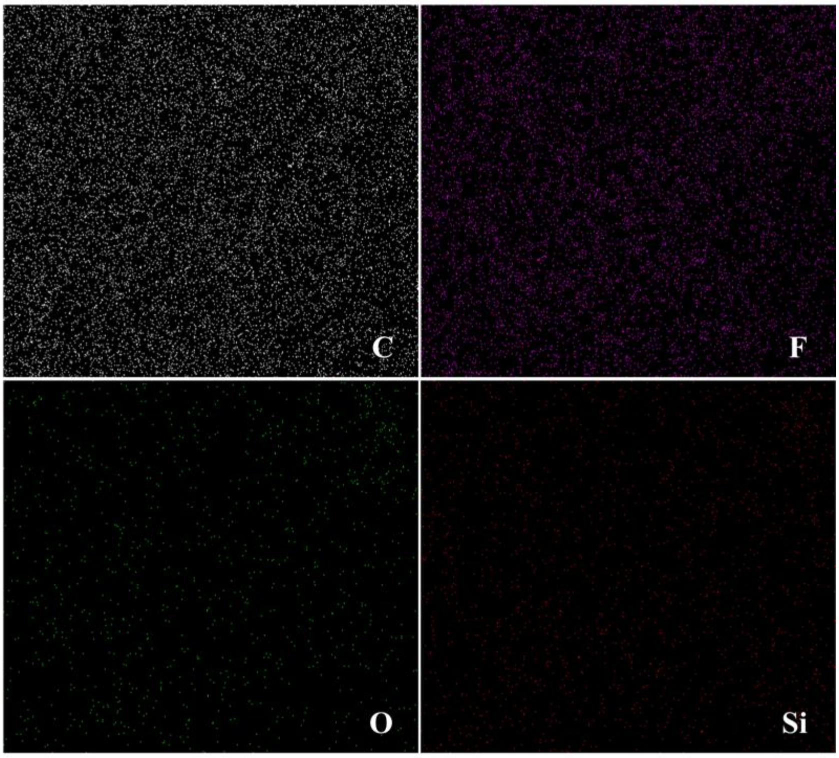

Element	Weigh/%	Atomic/%
C	45.76	57.16
O	2.04	1.91
F	51.06	40.32
Si	1.14	0.61
Totals	100.00	100.00

(b)

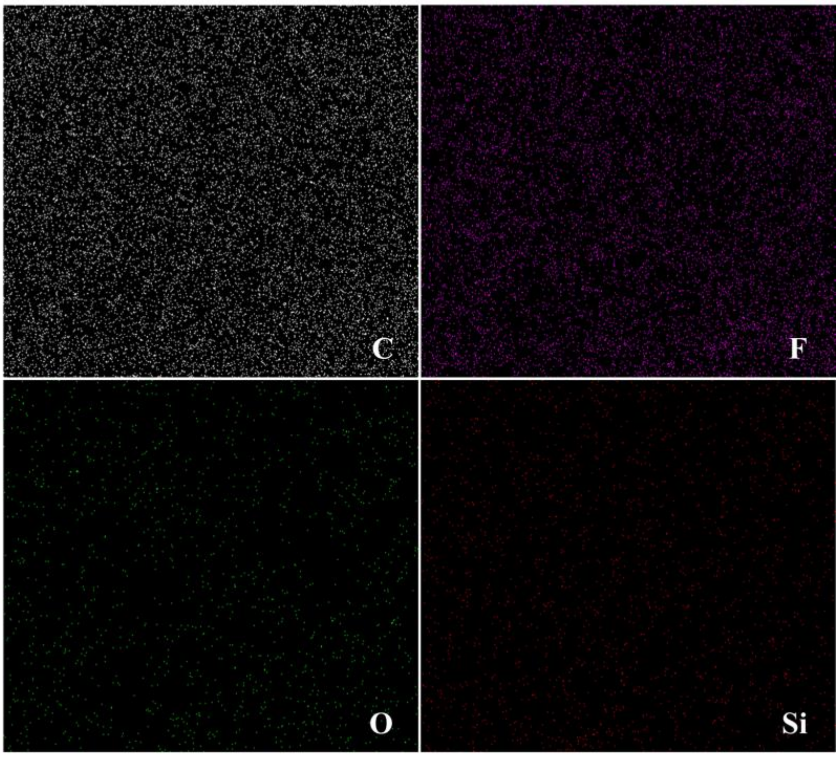

Element	Weigh/%	Atomic/%
C	47.21	58.55
O	2.40	2.29
F	49.24	38.61
Si	1.15	0.55
Totals	100.00	100.00

Figure S1. (a) Elemental analysis spectrograms of PHL membrane by EDS, (b) Elemental analysis spectrograms of PHP@PHL membrane by EDS.

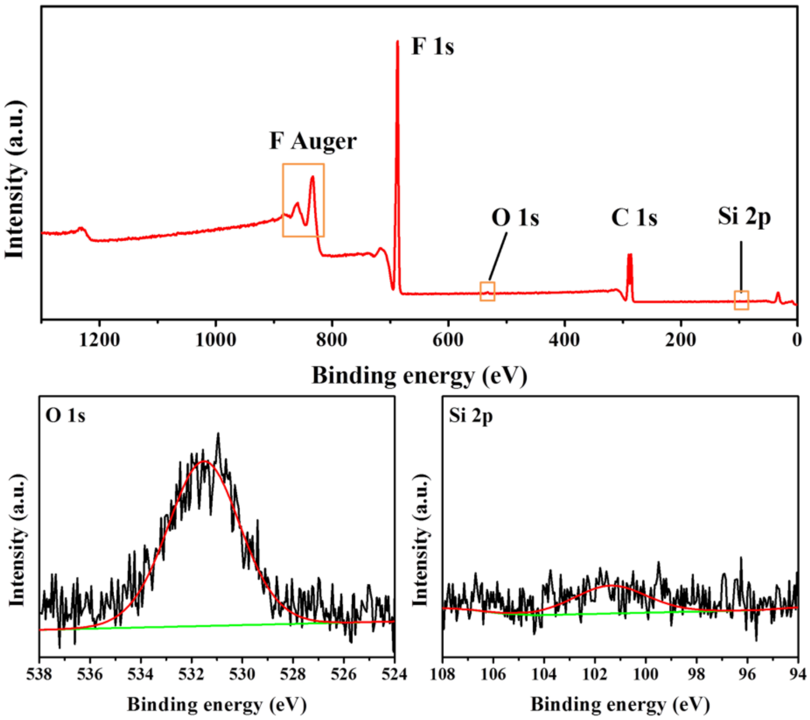

Element	C	O	F	Si	Cl
Atomic/%	47.52	0.42	51.87	0.19	0

Figure S2. XPS spectra of the PHP@PHL membrane.

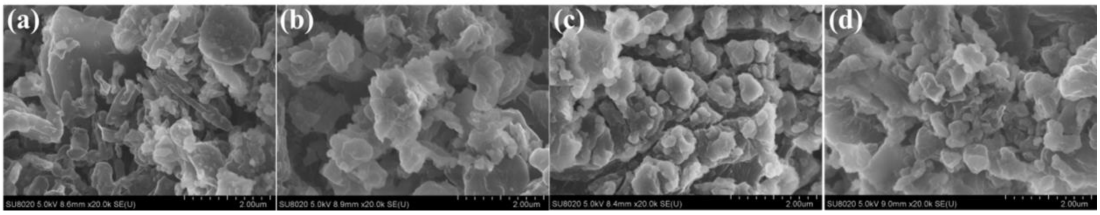

Figure S3. Morphology of the surface of lithium anode, (a) Celgard membrane in the liquid electrolyte after cycling for 300 h under a current density of 0.5mA/cm^2 ; (b) PVDF-HFP GPE, (c) PHL GPE, (d) PHP@PHL GPE after cycling for 1000 h under a current density of 0.5mA/cm^2.

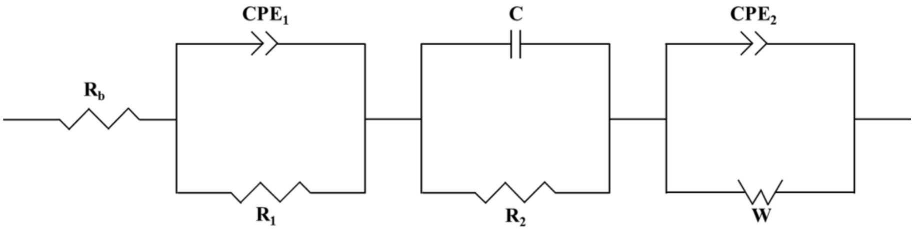

Figure S4. Equivalent circuit of the electrochemical impedance spectroscopy results in figure 6 (Li et al., 2013).

Table S1. Fitting data of the EIS results in figure 6.

Electrolytes	Days	R_b (Ω)	R_1(Ω)	R_2(Ω)
Celgard 2325	1	4.435	32.93	0.8115
	10	3.077	42.37	18.18
	20	4.902	57.75	22.46
	30	4.786	47.07	14.33
PVDF-HFP	1	3.588	21.35	1.675
	10	4.693	23.41	5.601
	20	2.921	23.46	9.234
	30	2.024	23.38	11.970
PHL	1	3.917	13.58	1.142
	10	4.239	12.68	2.043
	20	2.940	30.87	1.784
	30	3.792	33.75	1.674
PHP@PHL	1	8.859	15.71	1.255
	10	6.937	16.67	1.190
	20	3.374	18.46	1.947
	30	10.790	18.22	6.612

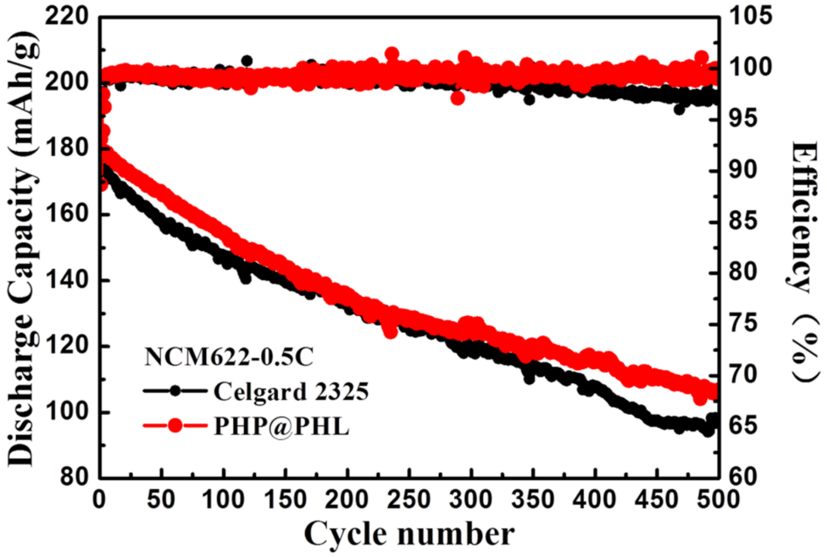

Figure S5. Cycling performance of the NCM622/Li cells with Celgard 2325 and PHP@PHL membrane at 0.5C.

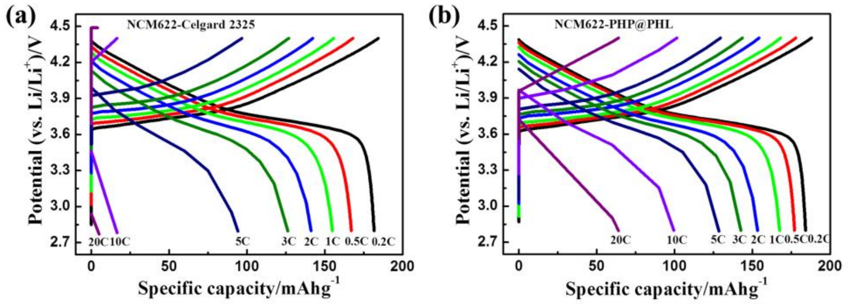

Figure S6. Charge-discharge voltage profiles of the NCM622/Li half-cells with (a) Celgard 2325 and (b) PHP@PHL membrane at different C rates.

Reference

Cheng, Y., Zhang, L., Xu, S., Zhang, H., Ren, B., Li, T., & Zhang, S. (2018). Ionic liquid functionalized electrospun gel polymer electrolyte for use in a high-performance lithium metal battery. *Journal of Materials Chemistry A, 6*(38), 18479-18487. doi:10.1039/c8ta06338a

Korf, K. S., Lu, Y., Kambe, Y., & Archer, L. A. (2014). Piperidinium tethered nanoparticle-hybrid electrolyte for lithium metal batteries. *J. Mater. Chem. A, 2*(30), 11866-11873. doi:10.1039/c4ta02219j

Li, L., Wang, J., Yang, P., Guo, S., Wang, H., Yang, X., Wu, B. (2013). Preparation and characterization of gel polymer electrolytes containing N-butyl-N-methylpyrrolidinium bis(trifluoromethanesulfonyl) imide ionic liquid for lithium ion batteries. *Electrochimica Acta, 88*, 147-156. doi:10.1016/j.electacta.2012.10.018

Lu, Y., Moganty, S. S., Schaefer, J. L., & Archer, L. A. (2012). Ionic liquid-nanoparticle hybrid electrolytes. *Journal of Materials Chemistry, 22*(9), 4066. doi:10.1039/c2jm15345a
